# Etiology and Clinical Manifestations of Acute Respiratory Infections in Pediatric Patients: A Retrospective Study in Moscow, Russia

**DOI:** 10.3390/diseases14090326

**Published:** 2026-09-06

**Authors:** Diana A. Grigoryan, Maria A. Gordukova, Elena V. Galeeva, Irina E. Turina, Ekaterina V. Ligskaya, Oleg B. Kovalev, Alina D. Matsvay

**Affiliations:** 1 Federal State Budgetary Institution “Centre for Strategic Planning and Management of Biomedical Health Risks” of the Federal Medical Biological Agency, Moscow 119121, Russia; dgrigoryan@cspfmba.ru; 2 G. Speransky Children’s Hospital No. 9, Department of Health of the City of Moscow, Moscow 123317, Russia; 3 N.F. Filatov Clinical Institute of Child Health, First I.M. Sechenov Moscow State Medical University (Sechenov University), Moscow 119991, Russialigskaya_e_v@staff.sechenov.ru (E.V.L.); 4 Institute of Maternal and Child Health, Russian National Research Medical University Named After N.I. Pirogov, Moscow 117997, Russia

**Keywords:** respiratory tract infections, virus diseases, morbidity, pediatrics, epidemiology

## Abstract

Background/Objectives: Acute respiratory viral infections (ARVIs) are the leading cause of pediatric morbidity and exhibit considerable clinical heterogeneity. The analysis of clinical and etiological associations, as well as the identification of factors linked to severe ARVI cases, is essential for effective epidemiological surveillance. Methods: A retrospective, single-center observational study was conducted including 1362 non-COVID-19 pediatric cases of acute respiratory infections (ARIs), registered between 2021 and 2024. Viral pathogens were detected using real-time PCR for broad-spectrum screening of respiratory viruses. Results: Viral etiology was established in approximately 50% of cases. The most frequently detected pathogen was Human rhinovirus (HRV) (35.6%), followed by Respiratory syncytial virus (RSV) and Human adenovirus (HAdV). Despite its predominance, HRV did not demonstrate nosological specificity. The strongest associations with lower respiratory tract infections were identified for RSV (OR = 3.2) and Human bocavirus (HBoV; OR = 2.4), predominantly within the pneumonia category. RSV was also associated with bronchitis/bronchiolitis (OR = 1.8). Significant associations with upper respiratory tract infections were established for Influenza viruses (OR = 10.4) and HAdV, including nasopharyngitis (OR = 3.88) and acute tonsillitis (OR = 3.9). HAdV additionally demonstrated an association with otitis media (OR = 5.1). Severe disease was registered in 52.3% of all clinical observations and occurred most frequently among young children, particularly in cases associated with respiratory syncytial virus (RSV >80%), as well as with human bocavirus (HBoV) and Human metapneumovirus (HMPV) (~55–59%). Co-infections were not associated with increased disease severity compared with monoinfections (OR = 1.1). Conclusions: The obtained results highlight the contribution of specific etiological agents to severe respiratory infections in children. These findings support the prioritization of such pathogens in clinical diagnostics, including the design of diagnostic algorithms and the selection of patient management strategies in pediatric populations.

## 1. Introduction

Acute respiratory infections (ARIs) remain a major challenge for healthcare systems due to the high transmissibility of their causative agents, rapid accumulation of genetic variations in viral genomes, extensive circulation within the population, and the presence of asymptomatic forms that facilitate undetected transmission. In the pediatric population, acute respiratory viral infections are most frequently detected in infants and preschool-aged children. In these age groups, the annual incidence reaches an average of 6–8 respiratory infection episodes per year, substantially exceeding the corresponding rates observed in adults (2–4 episodes annually) [1]. In addition, the duration of clinical manifestations in children generally averages approximately 14 days, whereas in adult patients the corresponding period is typically around 7 days [1].

In the Russian Federation, ARIs account for up to 90% of all infectious diseases and remain highly prevalent, with 27–41 million cases reported annually; children represent approximately 45–60% of all recorded episodes [2,3]. Official surveillance data for 2021–2024 demonstrate substantial year-to-year variation in ARI incidence; despite a gradual decline following the peak observed in 2022, the disease burden remained high, particularly among children younger than six years [4,5,6].

A major limitation of epidemiological surveillance for ARIs is the frequent occurrence of asymptomatic or subclinical infection. For example, in a large population-based study, more than 70% of infections caused by common respiratory viruses were shown to occur without pronounced clinical manifestations [7]. In a Russian screening study involving 274,000 individuals without ARI symptoms, genetic material of respiratory pathogens was detected in 6.1% of those examined, with rhinoviruses representing the most frequently identified agents [8]. Collectively, these data indicate a substantial contribution of latent forms of infection to respiratory virus circulation, considerably complicating epidemiological monitoring.

At the same time, even among patients with clinically verified ARI, the etiological agent frequently remains unidentified. Epidemiological observations indicate that up to 49–50% of ARI episodes remain without etiological confirmation despite the use of standard diagnostic algorithms, including molecular genetic techniques, are applied [9]. Molecular methods, particularly multiplex PCR, have significantly improved the detection of respiratory viruses enabling simultaneous testing for multiple pathogens with similar clinical presentations. However, a positive PCR result alone does not always confirm that the detected virus is the causative agent of the current illness, especially in cases of prolonged viral shedding or co-detection of several pathogens. The limited capacity for etiological verification of ARIs, associated with extensive viral heterogeneity and limitations of laboratory diagnostics, contributes to the formation of a “hidden” infectious reservoir, thereby reducing the informativeness of epidemiological surveillance and complicating the timely detection of emerging or atypical pathogens.

This study aims to investigate the correlations between the clinical manifestations of ARVIs and laboratory diagnostic data on respiratory viruses, in order to identify clinical and etiological associations as well as factors that determine the nosological form and severity of the disease.

## 2. Materials and Methods

### 2.1. Sample Collection

This retrospective single-center study was based on the analysis of molecular diagnostic data obtained from biological samples of pediatric patients with ARIs. The study size was determined by the number of observations meeting the eligibility criteria during the predefined study period; no a priori sample-size calculation was performed. The study was reported in accordance with the Strengthening the Reporting of Observational Studies in Epidemiology (STROBE) Statement (provided in Supplementary Table S5).

The samples were collected between 20 September 2021 and 31 July 2024, at the G. N. Speransky Children’s City Clinical Hospital No. 9. The study incorporated the results of PCR-based screening for viral pathogens performed as part of routine clinical practice. Molecular testing was prescribed for patients presenting clinical manifestations consistent with ARI, as well as for individuals with respiratory tract symptoms identified during medical evaluation or hospitalization for non-respiratory conditions.

Molecular analyses were performed using different types of biological specimens selected according to the patient’s clinical condition and the availability of the biological material. The study cohort was restricted to non-COVID-19 ARI cases; COVID-19 cases were not included in the dataset analyzed in the present study.

The study was conducted in accordance with the Declaration of Helsinki and approved by the Ethics Committee of G. N. Speransky Children’s Municipal Clinical Hospital No. 9 (protocol No. 39, 26 October 2021). Written informed consent was obtained from all participants or their legal representatives. All patient data were previously anonymized by replacing personal information with unique identification numbers. The metadata retained information on sex, age, specimen type, clinical department, and verified diagnosis, including disease category and ICD-11 classification code.

### 2.2. Molecular Genetic Testing

Molecular testing was performed using commercial reagent kits for nucleic acid extraction (“Ribo-prep”, AmpliTest, Moscow, Russia), reverse transcription (“Reverta-L”, AmpliTest, Moscow, Russia), and real-time PCR (RT-PCR), including “AmpliSens^®^ ARVI-screen-FL” (Moscow, Russia), “AmpliSens^®^ Influenza virus A/B-FL” (Moscow, Russia), and “AmpliSens^®^ Enterovirus-FL” (Moscow, Russia).

The assays were designed to detect Influenza A and B viruses (IV-A and IV-B), Human adenovirus (HAdV), Human bocavirus (HBoV), Respiratory syncytial virus (RSV), Human metapneumovirus (HMPV), human parainfluenza viruses, including Respirovirus types 1 and 3 (Human parainfluenza virus 1, hPIV-1; Human parainfluenza virus 3, hPIV-3) and Orthorubulavirus types 2 and 4 (Human parainfluenza virus 2, hPIV-2; Human parainfluenza virus 4, hPIV-4), Human rhinovirus (HRV), Enteroviruses (EV), and seasonal human coronaviruses (Human coronavirus OC43, 229E, NL63, and HKU1). The viral nomenclature used throughout the manuscript follows terminology commonly adopted in clinical and epidemiological literature and does not always correspond to the current taxonomic classification established by the International Committee on Taxonomy of Viruses.

Samples were classified as positive or negative according to cycle threshold (Ct) criteria specified in the manufacturer instructions for the corresponding diagnostic assay. Assay validity was controlled using positive, negative, and internal amplification controls included in each PCR test system. Both single-virus detections and cases involving simultaneous identification of multiple pathogens were included in the analysis.

### 2.3. Statistical Analysis of Associations

Associations between viral groups and clinical categories were assessed using approaches based on comparisons of observed and expected frequencies, as well as measures of relative association strength. Analyses were performed separately for two levels of clinical classification: ICD-11 disease categories and individual ICD-11 nosological forms.

For each “disease category–viral group” and “nosological form–viral group” pair, contingency tables were constructed in which the observed frequency O_ij_ corresponded to the number of cases with concurrent registration of the clinical category and pathogen group under consideration. As multiple clinical categories could be assigned to a single patient, the analysis was conducted at the level of “patient/sample–clinical category” pairs, with each pair treated as an independent binary observation for the corresponding clinical category.

The expected frequency E_ij_, assuming statistical independence between variables, was calculated as:

$$E_{ij}=c\frac{n_{i}\cdot n_{j}}{N},$$

where *n_i_* is the total number of observations within the i-th clinical category, *n_j_* represents the total number of detections of the j-th pathogen, and *N* corresponds to the overall sample size.

Global heterogeneity in the distribution of viral groups across clinical categories was assessed using the χ^2^ test of independence applied to the complete contingency matrices for “disease category–viral group” and “nosological form–viral group” combinations. In cases where statistically significant deviation from an independent distribution was identified, standardized Pearson residuals were additionally analyzed:

$$Z_{ij}=\frac{O_{ij}-E_{ij}}{\sqrt{E_{ij}}}$$

These values were used to evaluate the direction and magnitude of deviations of observed frequencies from expected frequencies. Positive *Z_ij_* values indicated observed frequencies exceeding expected levels, whereas negative values reflected reduced frequencies relative to expectation.

Observed and expected frequencies were also used to calculate the Lift metric:

$${Lift}_{ij}=\frac{O_{ij}}{E_{ij}}$$

To obtain a more symmetric distribution and facilitate visual comparison of enrichment magnitude, a log_2_(Lift) transformation was applied.

To assess individual pairwise associations, separate 2 × 2 contingency tables were constructed for each “clinical category–viral group” combination. These tables were used to calculate the χ^2^ statistic, *p*-values, and odds ratios (ORs) with 95% confidence intervals for all analyzed associations. ORs were calculated as:

$$OR=\frac{a\cdot d}{b\cdot c},$$

where *a*, *b*, *c*, and *d* represent the cell frequencies of the corresponding 2 × 2 contingency table. In the presence of zero counts, a continuity correction of 0.5 (Haldane-Anscombe correction) was applied to all cells of the respective table. Confidence intervals for ORs were calculated on the logarithmic scale as:

$$\ln\left(OR\right)\pm 1.96\times SE,SE=\sqrt{\frac{1}{a}+\frac{1}{b}+\frac{1}{c}+\frac{1}{d}},$$

using Woolf’s method, followed by back-transformation through exponentiation of the interval boundaries.

To account for multiple comparisons, *p*-values obtained from analyses of individual “clinical category–viral group” pairs were adjusted using the Benjamini–Hochberg procedure with calculation of q-values. Associations were considered statistically robust at *q* < 0.05. Results with *p* < 0.05 but *q* ≥ 0.05 were interpreted as nominally significant and were not regarded as confirmed following false discovery rate (FDR) correction. Complete calculation tables containing raw *p*-values and adjusted q-values are provided in the Supplementary Materials.

Unless otherwise specified, all ORs were reported as unadjusted estimates; multivariable adjustment for clinical covariates was not performed because detailed information on potential clinical confounders was not consistently available.

### 2.4. Analysis of Disease Severity

In the present analysis, severe disease was defined according to clinical and diagnostic criteria: admission to the intensive care unit (ICU), progression of respiratory infection to pneumonia, development of sepsis or septic shock, as well as respiratory failure grade III-IV and other severe complicated conditions documented in the clinical diagnosis.

The proportion of severe cases was calculated within each etiological group as the ratio of episodes with severe disease to the total number of detections of the corresponding pathogen, with 95% confidence intervals estimated using the Wilson score interval for binomial proportions.

Age was analyzed as a categorical variable using the following non-overlapping intervals: <1 month, ≥1 to <2 months, ≥2 to <6 months, ≥6 to <12 months, ≥1 to <5 years, ≥5 to <10 years, and ≥10 to ≤18 years. The same age categories were applied consistently throughout all age-stratified analyses, with finer stratification during the first year of life to preserve age-related resolution in infancy. Age data were unavailable for two observations; these cases were retained in analyses not involving age and excluded from age-stratified analyses. No imputation was performed.

Associations between age and severe disease were assessed using binary logistic regression, in which disease severity status was included as the dependent variable and age group as a categorical predictor; the 10–18-year age group served as the reference category. Model outputs were reported as ORs with 95% confidence intervals for each age category relative to the reference group.

For co-infection analysis, aggregated pairs of viral groups were generated, and the absolute number of observations together with the proportion of severe cases was calculated for each pair. In graphical representations of association and co-infection matrices, group size was additionally incorporated through marker scaling according to n; to reduce the disproportionate influence of highly represented categories, a nonlinear marker-size transformation (*n* = 0.5) was applied.

Computational data processing and graphical visualization were performed using the python (v3.12). The pandas library (v3.0.2) [10] was used for structuring, filtering, aggregation, and transformation of tabular data. Vectorized computations, including normalization procedures, calculation of derived statistical metrics, and mathematical transformations, were carried out using the NumPy package (v2.4.4) [11]. Statistical modeling was implemented using the statsmodels module (v0.14.6) [12].

## 3. Results

### 3.1. Characteristics of the Study Cohort

The analysis included 1362 ARI cases in pediatric patients registered between 20 September 2021 and 31 July 2024 not associated with COVID-19.

**Figure 1:**
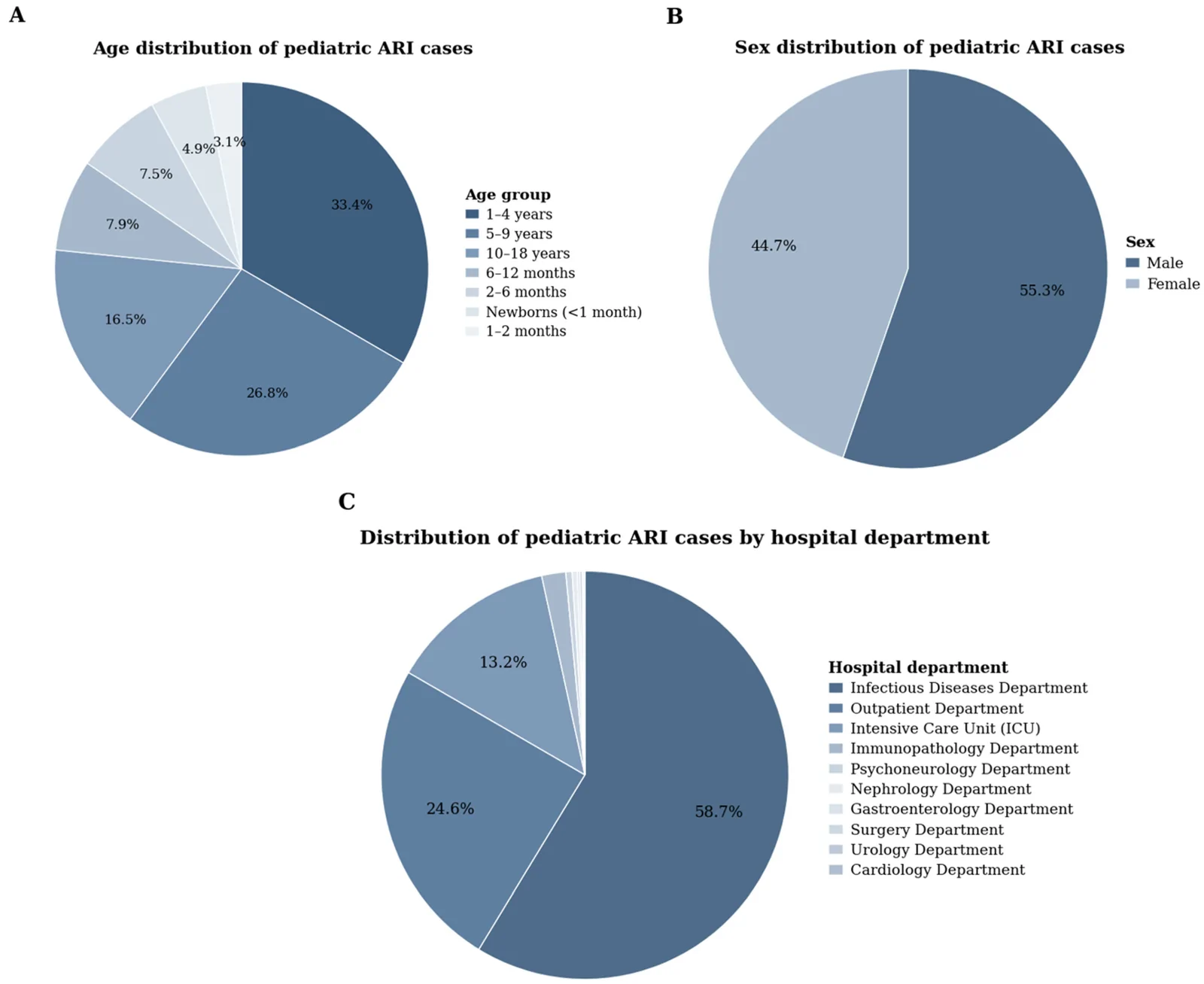
Characteristics of the study cohort of pediatric patients with ARIs. (**A**) Distribution of ARI cases across patient age groups; different color segments correspond to individual age categories according to the legend. (**B**) Distribution of ARI cases according to patient sex; color segments indicate the proportions of male and female patients. (**C**) Distribution of ARI cases across hospital clinical departments; color segments correspond to different clinical departments according to the legend. Percentages may not sum to 100% due to rounding.

The age structure of the cohort was characterized by a predominance of younger pediatric age groups. The largest proportion of patients belonged to the 1–4-year age group (33.38%), followed by the 5–9-year (26.76%) and 10–18-year (16.47%) groups. Patients aged <1 year accounted for 23.38% of the cohort, including newborns aged <1 month (4.85%), infants aged 1–2 months (3.09%), 2–6 months (7.50%), and 6–12 months (7.94%) (Figure 1A). The sex distribution was relatively balanced, with a slight predominance of male patients (55.3%) (Figure 1B).

Patient observations were registered across multiple clinical departments of the hospital. The majority originated from the infectious diseases department (n = 798; 58.63%), outpatient department (n = 335; 24.61%), and ICU (n = 180; 13.23%). Smaller numbers of cases were recorded in the immunopathology (n = 26; 1.91%), psychoneurology (n = 7; 0.51%), and nephrology (n = 6; 0.44%) departments. Isolated cases were documented in the gastroenterology, surgery, urology, and cardiology departments (Figure 1C). Overall, 75.4% observations were obtained from hospitalized patients.

In most cases, molecular testing was performed using oropharyngeal swab specimens (n = 1242; 91.19%). Less frequently used specimen types included tracheobronchial aspirates (n = 48; 3.52%), sputum samples (n = 22; 1.62%), material collected from endotracheal tubes (n = 18; 1.32%), pleural fluid (n = 16; 1.17%), and bronchoalveolar lavage samples (n = 14; 1.03%).

### 3.2. Analysis of Etiological Structure

**Figure 2:**
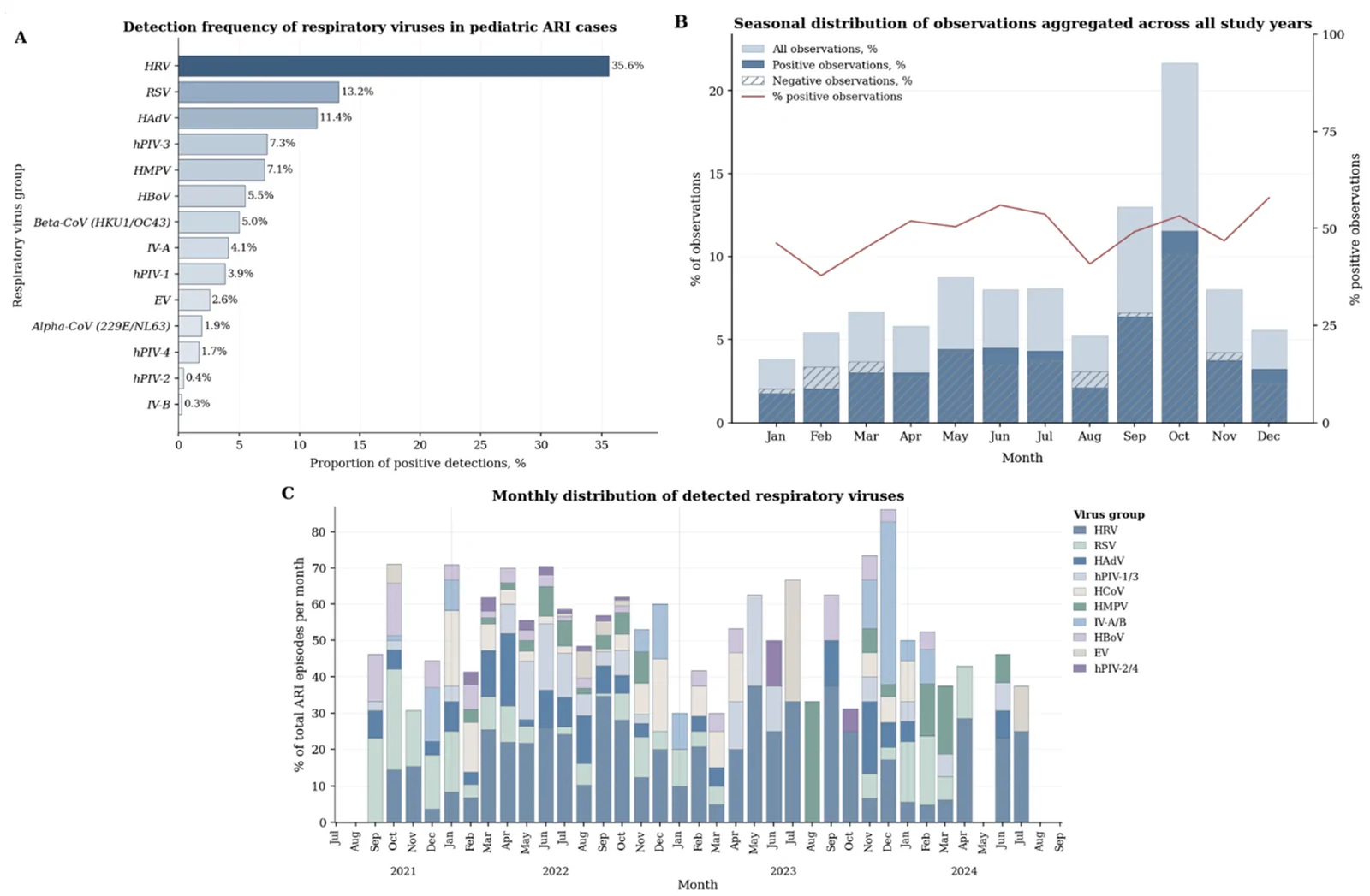
Etiological structure of respiratory viruses in the study cohort. (**A**) Distribution of detected respiratory viruses according to molecular diagnostic testing results; bar color intensity reflects the relative detection frequency, with proportional values calculated from the total number of virus-positive ARI episodes (n = 778). (**B**) Seasonal distribution of observations aggregated across all study years: light bars indicate all observations, dark bars indicate positive results, hatched bars indicate samples that tested negative for all viral targets included in the PCR panels used in this study, and the red line shows the proportion of laboratory-confirmed viral infections. (**C**) Monthly distribution of detected respiratory viruses throughout the study period; segment colors correspond to the major viral groups according to the legend, and segment height reflects their proportion among all ARI episodes recorded in the corresponding calendar month.

Molecular testing of the study cohort yielded a total of 778 positive detections of respiratory viruses (Figure 2A). The largest contribution to the detected pathogen spectrum was attributable to HRV (35.6%; n = 277). Substantial proportions were also represented by RSV (13.24%; n = 103), HAdV (11.44%; n = 89), hPIV-3 (7.33%; n = 57), and HMPV (7.07%; n = 55). Lower detection frequencies were observed for HBoV (5.53%; n = 43), Beta-CoV (HKU1/OC43) (5.0%; n = 39), IV-A (4.11%; n = 32), and hPIV-1 (3.86%; n = 30). The lowest detection rates were recorded for EV (2.57%; n = 20), Alpha-CoV (229E/NL63) (1.93%; n = 15), hPIV-4 (1.67%; n = 13), hPIV-2 (0.39%; n = 3), and IV-B (0.26%; n = 2).

For downstream analyses, detected viruses were aggregated into the following groups: HCoV (229E/NL63 and HKU1/OC43) was analyzed as a unified group of seasonal coronaviruses; hPIV-1 and hPIV-3 were combined into the Respirovirus group (hPIV-1/3); hPIV-2 and hPIV-4 into the Orthorubulavirus group (hPIV-2/4); and IV-A together with IV-B into the Influenza virus group (IV-A/B). Other pathogen groups, including HRV, RSV, HAdV, HMPV, HBoV, and EV, were analyzed without additional aggregation.

Analysis of monthly dynamics revealed a non-uniform distribution of patient observations with clinical manifestations of ARI throughout the study period. The highest numbers of registered cases were observed during the autumn months. The average proportion of laboratory-confirmed cases was approximately 50% (Figure 2B), reflecting the limited specificity of clinical diagnostics and the heterogeneous etiological structure of respiratory diseases.

Assessment of the monthly distribution of viral agents demonstrated pronounced variability in the etiological structure of ARIs across different calendar periods while maintaining persistent predominance of HRV throughout the observation period in the pediatric cohort. The highest absolute numbers of HRV detections were recorded in 2022, with peaks observed in September (n = 45) and October (n = 57). This period was also characterized by a broader spectrum of detected viral agents compared with subsequent years of observation. During the winter period, the detected pathogen spectrum was dominated by IV-A/B, HCoV, HRV, and RSV. The spring and summer months were characterized by predominance of HRV, HAdV, and hPIV-1/3. During the autumn period, HRV, RSV, and HAdV were detected most frequently (Figure 2C).

### 3.3. Associations Between Etiological Agents and Disease Categories

**Figure 3:**
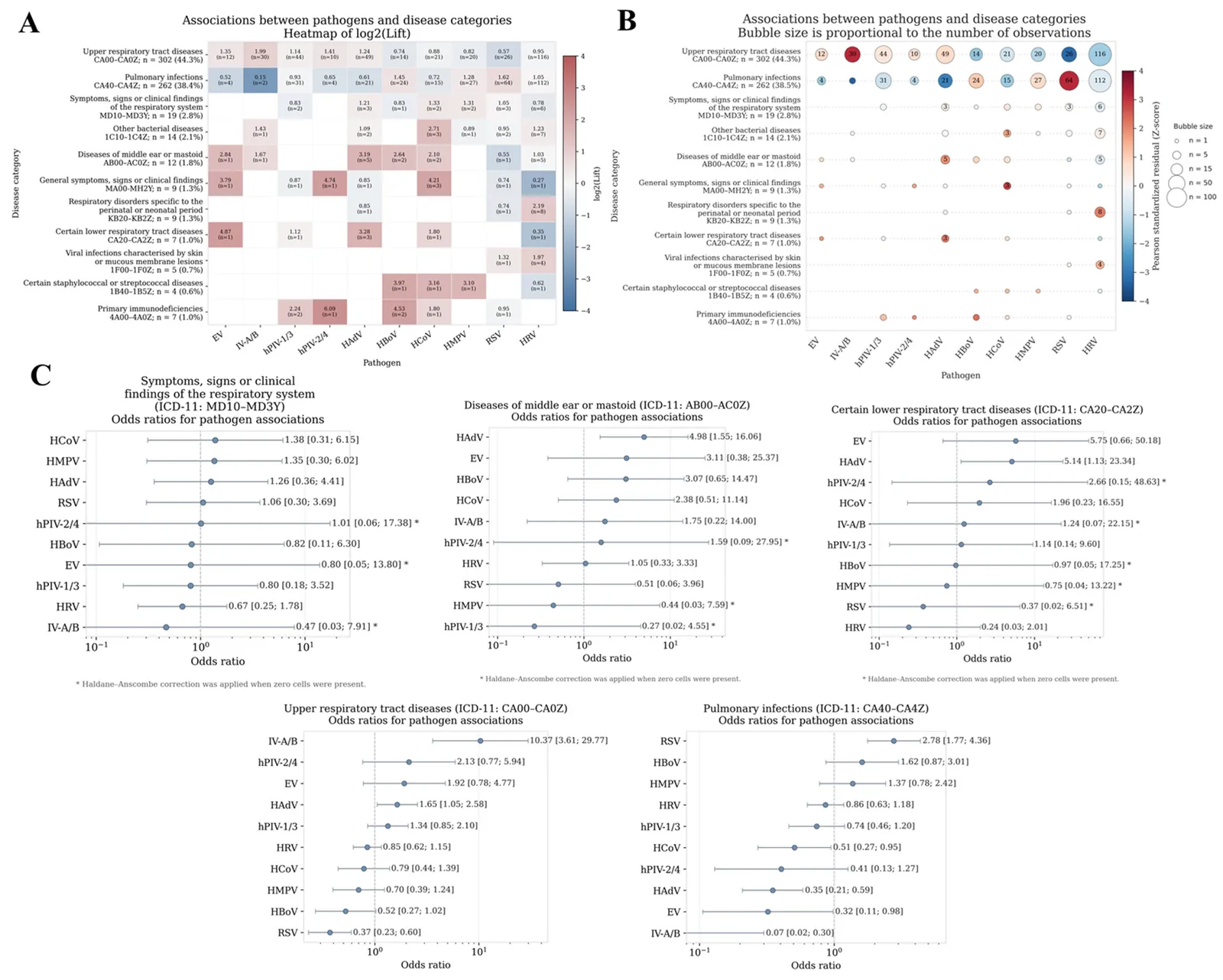
Associations between disease categories and viral groups. (**A**) Heatmap of log_2_(Lift) values reflecting deviations of the observed frequency of “disease category–viral group” associations from the expected frequency under the assumption of statistical independence. Values of log_2_(Lift) > 0 indicate overrepresentation of the corresponding association, whereas log_2_(Lift) < 0 indicate underrepresentation. The color scale represents log_2_(Lift), while the values displayed within the cells correspond to the original Lift values and the number of observations (n). (**B**) Bubble plot in which marker color corresponds to the Pearson standardized residuals (Z-scores) value and marker size reflects the number of observations (n); larger markers indicate higher numbers of registered associations, whereas the values displayed within the markers correspond to absolute observation counts. (**C**) Forest plot showing odds ratios (ORs) and 95% confidence intervals for associations between viral groups and individual ICD-11 disease categories; OR values > 1 indicate positive associations, whereas OR values < 1 indicate negative associations.

Analysis of the viral group distribution across nosological categories of respiratory diseases according to the ICD-11 classification revealed heterogeneous associations, as confirmed by consistent changes in the Lift-scores, standardized Pearson residuals, and ORs (Figure 3). Non-respiratory nosological categories were also included in the analysis when they were accompanied by clinical signs of respiratory tract involvement.

The most pronounced statistically significant association with the upper respiratory tract disease category (CA00–CA0Z) was observed for IV-A/B (Lift = 1.99, OR = 10.37, *p* < 0.001), corresponding to high positive standardized residuals (*Z* ≈ 3.8) and indicating a substantial excess over the expected detection frequency. For HAdV, a less pronounced positive association with this category was also observed (Lift = 1.24; OR = 1.65; *p* = 0.028); however, statistical significance was not maintained after correction for multiple comparisons (*q* > 0.05).

Within the pulmonary infection category (CA40–CA4Z), the strongest positive association was found for RSV (Lift = 1.62; OR = 3.16; *p* < 0.001), which corresponds to high Z-score values (up to 3.9) and indicates a stable association. HBoV also showed a tendency toward increased representation in the pulmonary infection category (Lift = 1.45; OR = 2.13; *p* = 0.015), although statistical significance was not retained following correction for multiple comparisons.

For the category “Symptoms, signs or clinical findings of diseases of the respiratory system” (MD10–MD3Y), no statistically significant associations with individual viral groups were identified. The corresponding metrics remained close to expected values (Lift ≈ 0.8–1.3; OR ≈ 1) in the absence of statistical significance (*p* > 0.3), consistent with low standardized residual values (∣Z∣ ≤ 1). In the category of middle ear diseases (AA00–AC0Z), HAdV was distinguished by a significant association (Lift = 3.19; OR = 4.98; *p* = 0.003), accompanied by positive Z-score (>2).

For most other categories, including general clinical symptoms, perinatal respiratory disorders, and selected infectious conditions, isolated deviations in Lift values were observed (exceeding 2–4 in some cases). However, these deviations occurred against a background of low expected frequencies, small sample counts, and in most cases did not reach statistical significance, which limits their interpretation as reproducible associations.

### 3.4. Associations Between Etiological Agents and Nosological Forms of Respiratory Infections

**Figure 4:**
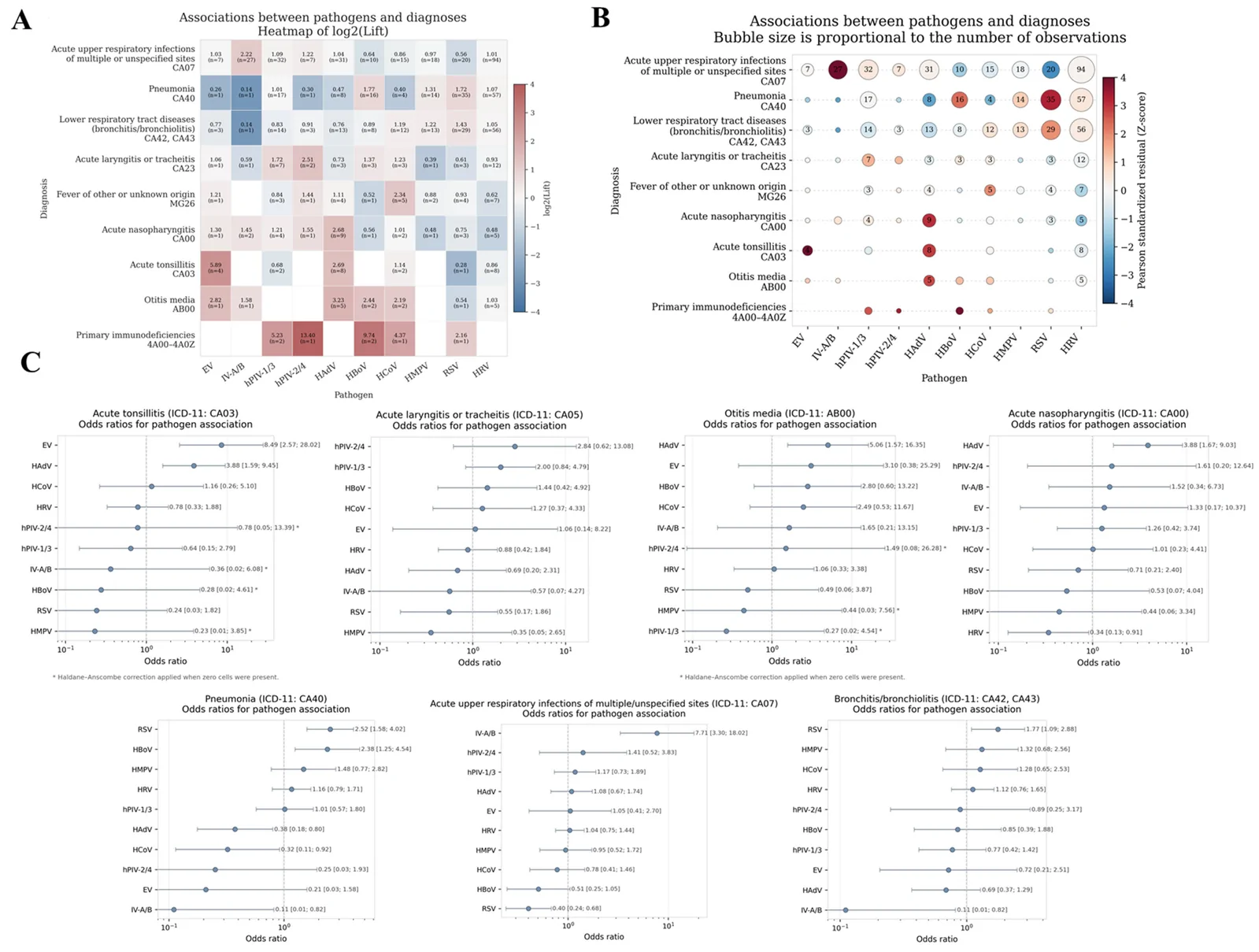
Associations between nosological forms and viral groups. (**A**) Heatmap of log_2_(Lift) values reflecting deviations of the observed frequency of “nosological form–viral group” associations from the expected frequency under the assumption of statistical independence. Values of log_2_(Lift) > 0 indicate overrepresentation of the corresponding association, whereas log_2_(Lift) < 0 indicate underrepresentation. The color scale represents log_2_(Lift), while the values displayed within the cells correspond to the original Lift values and the number of observations (*n*). (**B**) Bubble plot in which marker color corresponds to the Pearson standardized residuals (Z-score) value and marker size reflects the number of observations (*n*); larger markers indicate higher numbers of registered associations, whereas the values displayed within the markers correspond to absolute observation counts. (**C**) Forest plot showing odds ratios (ORs) and 95% confidence intervals for associations between viral groups and individual ICD-11 nosological forms; OR values > 1 indicate positive associations, whereas OR values < 1 indicate negative associations.

Analysis of associations at the level of individual clinical diagnoses identified a limited number of stable and statistically concordant associations (Figure 4). The strongest positive association was observed between influenza viruses and acute upper respiratory tract infections involving multiple or unspecified anatomical sites (CA07): the observed frequency substantially exceeded the expected value (Lift = 2.22; Z = 4.25), while the OR reached 7.71 (95% CI: 3.30–18.02; *p* = 4.9 × 10^−8^).

Within the pneumonia category (CA40), the most pronounced positive associations were identified for RSV and HBoV. For RSV, the corresponding values were Lift = 1.72 and *Z* = 3.26, with an OR of 2.52 (95% CI: 1.58–4.02; *p* = 7.2 × 10^−5^). HBoV likewise demonstrated an excess over the expected detection frequency (Lift = 1.77; *Z* = 2.32), with an OR of 2.38 (95% CI: 1.25–4.54; *p* = 0.007).

Among the analyzed viral groups, RSV showed the highest positive association with lower respiratory tract diseases (bronchitis/bronchiolitis; CA42–CA43), although the effect size remained moderate (Lift = 1.43, *Z* = 1.92; OR = 1.77, 95% CI: 1.09–2.88; *p* = 0.019).

At the level of individual upper respiratory tract nosological forms, the strongest association was found for adenoviruses in acute nasopharyngitis (CA00): Lift = 2.68, *Z* = 3.08, with an OR of 3.88 (95% CI: 1.67–9.03; *p* = 7.5 × 10^−4^). For acute tonsillitis (CA03), the strongest association was observed for enteroviruses, with Lift = 5.89 and Z = 4.03; the corresponding OR was 8.49 (95% CI: 2.57–28.02; *p* = 3.1 × 10^−5^). A similar, although less pronounced, association was identified for HAdV (Lift = 2.69, *Z* = 2.92, OR = 3.88; 95% CI: 1.59–9.45; *p* = 0.0014). Despite the magnitude of the observed effects, interpretation is limited by the relatively small number of observations (*n* = 4 for EV and *n* = 8 for HAdV) and should therefore be interpreted with considerable caution. For the remaining viral groups, no statistically significant associations were identified in this category.

In the nosological form of acute laryngitis or tracheitis (CA23), the highest effect estimates were observed for hPIV-2/4 (Lift = 2.51; *Z* = 1.35; OR = 2.84, 95% CI: 0.62–13.08) and hPIV-1/3 (Lift = 1.72; *Z* = 1.45; OR = 2.00, 95% CI: 0.84–4.79); however, these associations did not achieve statistical significance.

Additional positive associations were identified for HCoV in cases of fever of unknown origin (MG26) (Lift = 2.34; *Z* = 1.96; OR = 2.82, 95% CI: 1.02–7.78; *p* = 0.037) and for HAdV in cases of otitis media (AB00) (Lift = 3.23; *Z* = 2.77; OR = 5.06, 95% CI: 1.57–16.35; *p* = 0.0027). HBoV and HCoV also showed elevated Lift values (2.44 and 2.19, respectively) and OR estimates (2.8 and 2.49, respectively) in otitis media; however, statistical significance was not achieved in these cases.

### 3.5. Association Between Disease Severity and Etiology

**Figure 5:**
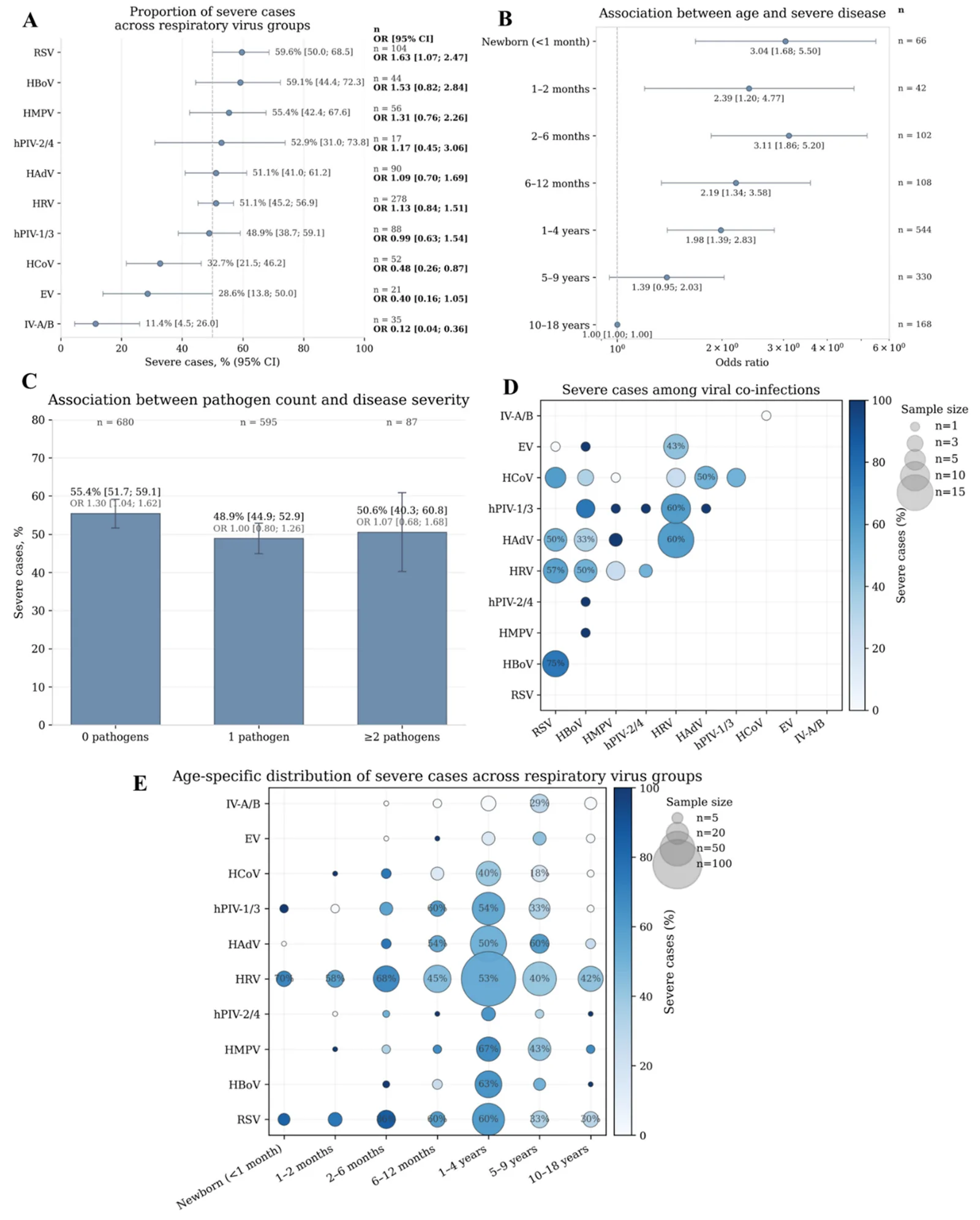
Associations of disease severity with viral groups, age, and viral co-infections. (**A**) Forest plot showing the proportion of severe cases (%) across different viral groups with 95% confidence intervals and corresponding OR values; points indicate point estimates, whereas horizontal lines indicate confidence intervals. (**B**) Forest plot illustrating the association between age groups and severe disease course; OR values were calculated relative to the 10–18 years reference group and are presented with 95% confidence intervals on a logarithmic scale. (**C**) Bar plot showing the proportion of severe cases according to the number of detected viral pathogens (0, 1, ≥2); bars indicate the proportion of severe cases, vertical lines show 95% confidence intervals, and OR values are given relative to the reference category “1 pathogen”. (**D**) Bubble plot showing the distribution of severe cases among pairs of viral co-infections; marker size is proportional to the number of observations, whereas marker color reflects the proportion of severe forms. (**E**) Bubble plot showing the distribution of severe cases across age and viral groups; marker size corresponds to the number of observations, whereas marker color represents the proportion of severe cases.

Within the overall ARI cohort, severe disease accounted for 52.3% of cases (712/1362). Analysis of severe disease distribution according to viral etiology revealed heterogeneity in the frequency of severe forms among different viral groups, as reflected by the corresponding proportions and OR values (Figure 5A).

The highest proportion of severe disease was observed for RSV at 59.6% (95% CI: 50.0–68.5), accompanied by an increased likelihood of severe outcome relative to other etiological agents (OR = 1.63; 95% CI: 1.07–2.47). Comparable proportions of severe cases were recorded for HBoV (59.1%; 95% CI: 44.4–72.3) and HMPV (55.4%; 95% CI: 42.4–67.6); however, the corresponding OR confidence intervals included unity (OR = 1.53; 95% CI: 0.82–2.84 and OR = 1.31; 95% CI: 0.76–2.26, respectively), indicating the absence of a statistically significant effect. For HRV and HAdV, the proportion of severe cases was 51.1% (95% CI: 45.2–56.9 and 41.0–61.2, respectively), with OR values close to 1 (HRV: 1.13; 95% CI: 0.84–1.51; HAdV: 1.09; 95% CI: 0.70–1.69), consistent with the absence of a pronounced association with disease severity.

In contrast, reduced proportions of severe disease were observed for HCoV and EV, reaching 32.7% (95% CI: 21.5–46.2) and 28.6% (95% CI: 13.8–50.0), respectively, accompanied by lower odds of severe disease (HCoV: OR = 0.48; 95% CI: 0.26–0.87; EV: OR = 0.40; 95% CI: 0.16–1.05). The lowest proportion of severe cases was recorded for IV-A/B at 11.4% (95% CI: 4.5–26.0), with a markedly reduced OR (0.12; 95% CI: 0.04–0.36).

### 3.6. Association Between Disease Severity and Age

The distribution of severe cases demonstrated a pronounced age dependence. The highest proportions of severe disease were observed in the youngest age groups: 65.2% (95% CI: 53.1–75.5) among neonates (<1 month) and 65.7% (95% CI: 56.1–74.2) among children aged 2–6 months. With increasing age, a progressive decline in disease severity was observed: 57.4% in the 6–12-month group, 55.0% in children aged 1–4 years, 46.1% in the 5–9-year group, and 38.1% among 10–18-year group (Figure 5B).

Stratified analysis by viral group and age demonstrated that RSV was associated with persistently high proportions of severe cases predominantly in early childhood, reaching 83.3% among neonates and 85.7% in the 2–6-month age group, followed by a decline to 30–33% in older children. Comparably high severity estimates in younger age groups were observed for HBoV (100% in the 2–6-month group, underrepresented subgroup, *n* = 2; 63.3% in the 1–4-year age group), as well as for HMPV (up to 66.7% both in the 6–12-month and the 1–4-year age groups). However, substantial variability in severity estimates across age groups was observed for HBoV and HMPV, partially attributable to the limited sample size of individual subgroups (Figure 5E).

### 3.7. Association Between Disease Severity and Co-Infections

Analysis of the relationship between disease severity and the number of detected pathogens showed that in cases without viral detection by the diagnostic panels, the proportion of severe disease was 55.4% (95% CI: 51.7–59.1), whereas in cases with detection of a single pathogen the corresponding proportion was 48.9% (95% CI: 44.9–52.9). For co-infections (≥2 pathogens), the proportion of severe cases reached 50.6% (95% CI: 40.3–60.8). No statistically significant differences from the group with a single pathogen were identified for co-infections (OR = 1.07; 95% CI: 0.68–1.68) (Figure 5C).

The structure of co-infections was characterized predominantly by the absence of pronounced deviations from random distribution of viral combinations (Figure 5D). For most viral pairs, Lift values remained close to 1, while standardized Pearson residuals (Z-scores) ranged from −2 to +2, indicating no statistically significant deviation from expected frequencies. Pair-specific deviations were not interpreted further because temporal variation in virus circulation was not accounted for in this analysis.

## 4. Discussion

Among the identified viral groups, HRV demonstrated the highest prevalence characterized by broad nosological heterogeneity. HRV was detected across multiple clinical forms, including upper respiratory tract infections (URTIs), bronchitis, pneumonia, laryngotracheitis, tonsillitis, and nasopharyngitis. This distribution indicates the nosological non-specificity of HRV within the pediatric cohort. However, given the predominance of HRV detections in the study population, its contribution to the structure of individual nosological categories should be interpreted with caution, as the high representation may primarily reflect epidemiological predominance within the cohort rather than selective association with specific nosological forms.

Alongside HRV, RSV made a substantial contribution to the structure of detected pathogens. RSV is traditionally regarded as one of the leading causative agents of respiratory infections in children, particularly in the context of lower respiratory tract infections (LRTIs). The present findings were consistent with data from clinical and epidemiological studies conducted across several European countries, in which RSV and HRV are considered the main viral agents determining the pattern of pediatric respiratory morbidity [13,14,15]. Partial agreement was also observed in comparison with Russian epidemiological surveillance data from 2024, where HRV and RSV similarly occupied leading positions among non-influenza respiratory viruses (22% and 13%, respectively), despite the overall predominance of influenza viruses within the structure of seasonal respiratory morbidity (23%) [5]. At the same time, direct comparison was limited by differences in the age composition of the analyzed cohorts, as population-based surveillance data encompass all age groups, whereas the present study was conducted exclusively in a pediatric cohort consisting predominantly of children younger than 10 years.

Influenza infection in pediatric practice is characterized by clinical heterogeneity and may manifest as involvement of either the upper or lower respiratory tract. The association between influenza viruses and upper respiratory tract infections identified in the present study may reflect features of the early clinical manifestation of the disease, which typically includes fever, cough, rhinitis, and pharyngitis. At the same time, data from large epidemiological studies indicate a substantial contribution of influenza viruses to the structure of LRTI. Thus, both the 2011 meta-analysis involving children younger than 5 years of age (43 studies and global epidemiological data) [16] and the subsequent updated global burden analysis published in 2018 (based on more than 150 data sources) [17] classified influenza viruses among the major causes of acute LRTIs and hospitalizations in children.

The observed clinical association between adenoviral infection and URTIs was consistent with current understanding of adenovirus pathogenesis in children. HAdV exhibits pronounced tropism for respiratory epithelium and lymphoid tissue of the oropharynx, contributing to its substantial role in the development of the clinical presentation of pharyngotonsillitis. For example, in a Spanish clinical study involving 209 children with adenoviral infection, tonsillitis was identified in up to 88% of cases, while more than half of the patients exhibited exudative tonsillar inflammation, frequently mimicking a bacterial process [18]. In a recent clinical review (Italy, 2025) HAdV was likewise described as one of the leading viral causes of pharyngotonsillitis and upper respiratory tract infections in children [19].

The association between HAdV and middle ear pathology identified in the present study was also consistent with published clinical data. In American prospective study involving 294 children aged 6 months to 3 years, adenovirus-associated upper respiratory tract infections were shown to be characterized by an increased frequency of acute otitis media as a complication [20].

Despite the high representation of HRV among virus-positive pneumonia cases, a more pronounced association with this nosological form was observed for RSV, which is consistent with its established role as one of the principal causative agents of acute LRTIs in children. According to a large meta-analysis including 186 studies and more than 152,000 children with community-acquired pneumonia, RSV and HRV were among the most frequently detected viral agents [21]. The association between HBoV and pneumonia is also of clinical interest and is consistent with data implicating this virus in the development of acute LRTIs, particularly in the context of co-infections [22].

A similar pattern was observed for other forms of LRTIs, particularly bronchitis and bronchiolitis, in which RSV likewise retained a leading etiological role. This aligns with the current understanding of RSV pathogenesis, characterized by pronounced tropism for the epithelium of the lower respiratory tract and high clinical relevance in young children [23]. The contribution of other respiratory viruses, including HMPV and seasonal coronaviruses, to the development of these nosological forms appears less pronounced and is likely to depend more substantially on patient age and clinical context.

Severe forms of ARI accounted for more than half of all cases in the analyzed pediatric cohort. This proportion should be interpreted in the context of the hospital-based study population, which was inherently skewed toward clinically significant disease due to the inclusion of predominantly hospitalized patients. Furthermore, the 2021–2024 study period overlapped with pandemic and post-pandemic changes in respiratory virus circulation and hospitalization patterns, which may have further influenced the clinical composition of the cohort [24].

Analysis of the etiological structure of severe cases revealed considerable heterogeneity, with the most stable association with severe disease observed for RSV. HBoV and HMPV also showed a tendency toward association with severe forms of ARI. These findings align with previously published clinical and epidemiological data. According to global epidemiological studies and meta-analysis of global data, RSV and HMPV are among the major viral agents associated with severe lower respiratory tract infections and hospitalization in children, including previously healthy pediatric patients [25,26].

Published data likewise supports a clinical association between HBoV and complicated forms of ARI. In a Japanese epidemiological study, 33% of hospitalized children with HBoV1 monoinfection required admission to the intensive care unit, while 67% required mechanical ventilation [27]. In another prospective study, the frequency of mechanical ventilation in bocavirus infection reached 52%, and the mean duration of hospitalization was 31.4 days [28].

In contrast, seasonal coronaviruses, enteroviruses, and influenza viruses were less strongly associated with severe forms of ARI in the analyzed cohort, which may reflect differences in the pathogenic mechanisms and clinical course of the infections they cause.

The age-related severity profile of ARIs in the pediatric cohort was characterized by predominance of severe forms among children during the first months of life, followed by a gradual decline in severity frequency in older age groups. This pattern is in agreement with current understanding of the increased susceptibility of young children to severe respiratory infections, which is commonly attributed to functional immaturity of the immune response and anatomical and physiological features of the respiratory tract [29]. This age dependence was observed most consistently for RSV, in agreement with the established role of RSV as a leading contributor to severe LRTIs in early age [30]. Comparable severity estimates in younger age groups were also observed for HBoV and HMPV, although these viral agents were characterized by greater variability in statistical indicators.

In the analyzed cohort, the presence of co-infection was not associated with increased clinical severity compared with monoinfections, indicating no overall association between multiple viral detection and disease severity. However, published data suggest that the clinical impact of co-infection may vary depending on the specific combination of viruses. In a systematic review and meta-analysis of pediatric cohorts involving children under 5 years of age with RSV infection, no significant increase in severity was identified for co-infections compared with RSV-monoinfection, except for RSV-HMPV co-infection, which was associated with an increased risk of ICU admission [31]. A similar dependence on the specific viral combination was observed in a multicenter clinical study conducted in Lombardy, Italy [32], including 1788 children under 2 years of age with bronchiolitis, where co-infections not involving RSV were associated with a more favorable clinical course, whereas the combinations of RSV with other viruses were accompanied by a higher probability of pneumonia development compared with RSV monoinfection [33]. Thus, although co-infection per se was not associated with increased disease severity in the present cohort, individual viral combinations may have distinct clinical effects and warrant further investigation.

The results of the present study should be interpreted with consideration of several methodological limitations. The retrospective single-center design restricts the extrapolation of the obtained findings to broader populations and does not allow comprehensive adjustment for potential confounding factors, including comorbidities, prior therapy, vaccination status, and timing of specimen collection. Clinicopathological associations were evaluated using diagnoses documented in the available medical records and categorized according to ICD-11. However, detailed information on clinically relevant host factors, including prematurity, congenital heart disease, asthma, and other underlying condition, was not consistently available and could not be incorporated into the analysis. Another important factor relates to patient selection for PCR diagnostics, as molecular testing was performed predominantly in hospitalized patients presenting with clinically significant ARI symptoms. Accordingly, the observed etiological distribution and severity profile may not be directly generalizable to community-based pediatric populations or to healthcare settings with different diagnostic and hospitalization protocols. The retrospective design precluded quantitative assessment of the magnitude of these potential biases.

Interpretation of the identified clinic–pathological associations also requires consideration of the cohort structure, which was dominated by children from younger age groups (1–4 years) characterized by increased susceptibility to respiratory infections. Age-related characteristics of the immune response, immaturity of respiratory tract defense mechanisms, and high contact intensity within organized groups may contribute both to increased infection frequency and to variability in clinical course.

It should also be taken in account that only approximately half of the analyzed specimens were accompanied by laboratory detection of a viral agent. Given the use of multiplex PCR systems, this observation likely reflects the multifactorial nature of clinical ARI cases and may be attributable to the involvement of viruses not included in the diagnostic panel, bacterial etiology in a subset of episodes, as well as delayed specimen collection in the setting of declining viral load. Additionally, the predominance of oropharyngeal swab specimens should be considered a pre-analytical limitation, as specimen type may influence molecular detection sensitivity and, consequently, the proportion of PCR-negative observations. The absence of comparable bacteriological or serological verification limits the etiological interpretation of the findings, particularly in cases of co-detection and negative test results. Overall, the observed patterns reflect the structure of detectable rather than all circulating respiratory viruses, which should be considered when comparing the results with data from other studies.

## 5. Conclusions

The obtained results indicate the presence of distinct patterns in the distribution of the etiological profile across nosological forms of ARIs within the pediatric population. The most pronounced clinic–pathological associations within the structure of LRTIs were observed for RSV, predominantly in the context of pneumonia, bronchitis, and bronchiolitis, whereas pneumonia represented the prevailing nosological form associated with HBoV. URTIs were characterized by a more uniform pathogen distribution profile, with predominance of associations involving IV-A/B and HAdV. Additional clinically relevant associations included the relationship of HAdV with nasopharyngitis, acute tonsillitis, and otitis media. Disease severity was determined primarily by patient age and viral etiology, with the most unfavorable clinical profile observed among young children, particularly in cases associated with RSV infection, as well as infections involving HBoV and HMPV. Collectively, the obtained data expands current understanding of the etiology and clinical manifestations of pediatric ARIs and may contribute to improved assessment of the risk of severe disease course.

## Data Availability

Data is contained within the article or Supplementary Materials.

## Supplementary Materials

The following supporting information can be downloaded at: https://www.mdpi.com/article/10.3390/diseases14090326/s1, Table S1: Results of the global *χ^2^* test of independence for complete “clinical category-pathogen” contingency matrices; Table S2: Statistical parameters of the local association analysis between disease categories and viral groups based on 2 × 2 contingency tables; Table S3: Statistical parameters of the local association analysis between nosological forms and viral groups based on 2 × 2 contingency tables; Table S4: ICD-11 Diagnoses Included in the Disease Categories Used for Association Analysis; Table S5: Strengthening the Reporting of Observational Studies in Epidemiology (STROBE) Statement.

## Abbreviations

The following abbreviations are used in this manuscript:

ARI	Acute respiratory infection
ARVIs	Acute respiratory viral infections
BAL	Bronchoalveolar lavage
Beta-CoV	Betacoronavirus (HKU1/OC43)
CI	Confidence interval
Ct	Cycle threshold
EV	Enterovirus
FDR	False discovery rate
HAdV	Human adenovirus
HBoV	Human bocavirus
HCoV	Human coronavirus
HMPV	Human metapneumovirus
HRV	Human rhinovirus
hPIV	Human parainfluenza virus
hPIV-1/3	Human parainfluenza virus types 1 and 3 (Respirovirus group)
hPIV-2/4	Human parainfluenza virus types 2 and 4 (Orthorubulavirus group)
ICD-11	International Classification of Diseases, 11th Revision
ICU	Intensive care unit
IV-A/B	Influenza A and B viruses
Lift	Association enrichment metric calculated as observed/expected frequency ratio
LRTI	Lower respiratory tract infection
OR	Odds ratio
PCR	Polymerase chain reaction
q-value	Adjusted *p*-value after Benjamini–Hochberg correction
RSV	Respiratory syncytial virus
RT-PCR	Real-time polymerase chain reaction
URTI	Upper respiratory tract infection
Z-score	Standardized Pearson residual
