# Supplementary material for: Etiology and Clinical Manifestations of Acute Respiratory Infections in Pediatric Patients: A Retrospective Study in Moscow, Russia
Source: Diseases. 2026 Sep 6;14(9):326. doi: 10.3390/diseases14090326 (PMC13605983; doi:10.3390/diseases14090326)
Supplement: Supplementary file 1 [file diseases-14-00326-s001.zip › Supplementary material Tables S1-S4.pdf]

Supplementary materials

Table S1. Results of the global χ^2 test of independence for complete “clinical category-pathogen” contingency matrices.

Contingency matrix	Number of clinical categories (n)	Number of viral groups (n)	χ^2 value	Degrees of freedom (df)	p-value
Disease category / viral group	11	10	144,31	90	0,00029
Nosological form / viral group	11	10	180,8	90	$4{,}66\times10^{-8}$

Table S2. Statistical parameters of the local association analysis between disease categories and viral groups based on 2×2 contingency tables.

Disease categories	Viral group	a	b	c	d	Expected count	Lift	Risk ratio (RR)	Odds ratio (OR)	χ^2	p-value	q-value (Benjamini-Hochberg)
Upper respiratory tract diseases (CA00-CA0Z)	EV	12	8	290	372	8,86	1,35	1,37	1,92	2,06	0,15089	0,89634
	IV-A/B	30	4	272	376	15,06	1,99	2,10	10,37	28,02	1,20E-07	0,00002
	hPIV-1/3	44	43	258	337	38,52	1,14	1,17	1,34	1,60	0,20581	0,89634
	hPIV-2/4	10	6	292	374	7,09	1,41	1,43	2,13	2,20	0,13764	0,89634
	HAdV	49	40	253	340	39,41	1,24	1,29	1,65	4,82	0,02819	0,34567
	HBoV	13	30	289	350	19,04	0,68	0,67	0,52	3,67	0,05536	0,61084
	HCoV	21	33	281	347	23,91	0,88	0,87	0,79	0,69	0,40576	0,89634
	HMPV	20	35	282	345	24,35	0,82	0,81	0,70	1,52	0,21760	0,89634
	RSV	26	77	276	303	45,61	0,57	0,53	0,37	17,82	0,00002	0,00194
	HRV	116	161	186	219	122,6	0,95	0,91	0,85	1,09	0,29584	0,89634
Lung infections (CA40-CA4Z)	EV	4	16	258	404	7,68	0,52	0,51	0,39	2,95	0,08567	0,78331
	IV-A/B	2	32	260	388	13,06	0,15	0,15	0,09	16,01	0,00006	0,00403
	hPIV-1/3	31	56	231	364	33,42	0,93	0,92	0,87	0,33	0,56758	0,89634
	hPIV-2/4	4	12	258	408	6,15	0,65	0,65	0,53	1,25	0,26421	0,89634
	HAdV	21	68	241	352	34,19	0,61	0,58	0,45	9,50	0,00205	0,05469
	HBoV	24	19	238	401	16,52	1,45	1,50	2,13	5,87	0,01539	0,21410
	HCoV	15	39	247	381	20,74	0,72	0,71	0,59	2,81	0,09394	0,81247
	HMPV	27	28	235	392	21,13	1,28	1,31	1,61	2,88	0,08961	0,79656
	RSV	64	39	198	381	39,57	1,62	1,82	3,16	28,85	7,81E-08	0,00002
	HRV	112	165	150	255	106,4	1,05	1,09	1,15	0,80	0,37051	0,89634
Symptoms, signs or clinical findings of diseases of the respiratory system (MD10-MD3Y)	EV	0	20	19	643	0,56	0,00	0,81	0,80	0,59	0,44224	0,89634
	IV-A/B	0	34	19	629	0,95	0,00	0,48	0,47	1,03	0,31122	0,89634
	hPIV-1/3	2	85	17	578	2,42	0,83	0,80	0,80	0,09	0,76757	0,89634
	hPIV-2/4	0	16	19	647	0,45	0,00	1,01	1,01	0,47	0,49320	0,89634
	HAdV	3	86	16	577	2,48	1,21	1,25	1,26	0,13	0,71918	0,89634
	HBoV	1	42	18	621	1,20	0,83	0,83	0,82	0,04	0,84970	0,89634
	HCoV	2	52	17	611	1,50	1,33	1,37	1,38	0,18	0,66933	0,89634
	HMPV	2	53	17	610	1,53	1,31	1,34	1,35	0,16	0,68938	0,89634
	RSV	3	100	16	563	2,87	1,05	1,05	1,06	0,01	0,93242	0,93534
	HRV	6	271	13	392	7,72	0,78	0,67	0,67	0,66	0,41594	0,89634
Other bacterial diseases (1C10-1C4Z)	EV	0	20	14	648	0,41	0,00	1,09	1,09	0,43	0,51109	0,89634
	IV-A/B	1	33	13	635	0,70	1,43	1,47	1,48	0,14	0,70782	0,89634
	hPIV-1/3	0	87	14	581	1,79	0,00	0,23	0,23	2,09	0,14827	0,89634
	hPIV-2/4	0	16	14	652	0,33	0,00	1,35	1,36	0,34	0,55788	0,89634

Disease categories	Viral group	a	b	c	d	Expected count	Lift	Risk ratio (RR)	Odds ratio (OR)	χ^2	p-value	q-value (Benjamini-Hochberg)
	HAdV	2	87	12	581	1,83	1,09	1,11	1,11	0,02	0,88968	0,90380
	HBoV	0	43	14	625	0,88	0,00	0,50	0,50	0,96	0,32672	0,89634
	HCoV	3	51	11	617	1,11	2,71	3,17	3,30	3,58	0,05853	0,62433
	HMPV	1	54	13	614	1,13	0,89	0,88	0,87	0,02	0,89817	0,90954
	RSV	2	101	12	567	2,11	0,95	0,94	0,94	0,01	0,93126	0,93534
	HRV	7	270	7	398	5,69	1,23	1,46	1,47	0,52	0,47005	0,89634
Diseases of the middle ear or mastoid process (AA00-AC0Z)	EV	1	19	11	651	0,35	2,84	3,01	3,11	1,25	0,26324	0,89634
	IV-A/B	1	33	11	637	0,60	1,67	1,73	1,75	0,29	0,59083	0,89634
	hPIV-1/3	0	87	12	583	1,53	0,00	0,27	0,27	1,79	0,18141	0,89634
	hPIV-2/4	0	16	12	654	0,28	0,00	1,57	1,59	0,29	0,58802	0,89634
	HAdV	5	84	7	586	1,57	3,19	4,76	4,98	8,82	0,00299	0,06842
	HBoV	2	41	10	629	0,76	2,64	2,97	3,07	2,22	0,13623	0,89634
	HCoV	2	52	10	618	0,95	2,10	2,33	2,38	1,28	0,25747	0,89634
	HMPV	0	55	12	615	0,97	0,00	0,45	0,44	1,07	0,30061	0,89634
	RSV	1	102	11	568	1,81	0,55	0,51	0,51	0,44	0,50879	0,89634
	HRV	5	272	7	398	4,87	1,03	1,04	1,05	0,01	0,94039	0,94039
General symptoms, signs or clinical findings (MA00-MH2Y)	EV	1	19	8	654	0,26	3,79	4,14	4,30	2,14	0,14321	0,89634
	IV-A/B	0	34	9	639	0,45	0,00	0,98	0,98	0,48	0,48909	0,89634
	hPIV-1/3	1	86	8	587	1,15	0,87	0,85	0,85	0,02	0,88159	0,89843
	hPIV-2/4	1	15	8	658	0,21	4,74	5,20	5,48	3,06	0,08032	0,75595
	HAdV	1	88	8	585	1,17	0,85	0,83	0,83	0,03	0,86201	0,89634
	HBoV	0	43	9	630	0,57	0,00	0,77	0,76	0,61	0,43339	0,89634
	HCoV	3	51	6	622	0,71	4,21	5,81	6,10	8,08	0,00448	0,09467
	HMPV	0	55	9	618	0,73	0,00	0,59	0,59	0,80	0,37108	0,89634
	RSV	1	102	8	571	1,36	0,74	0,70	0,70	0,11	0,73638	0,89634
	HRV	1	276	8	397	3,66	0,27	0,18	0,18	3,29	0,06963	0,71296
Respiratory disorders specific to the perinatal or neonatal period (KB20-KB2Z)	EV	0	20	9	653	0,26	0,00	1,66	1,68	0,28	0,59964	0,89634
	IV-A/B	0	34	9	639	0,45	0,00	0,98	0,98	0,48	0,48909	0,89634
	hPIV-1/3	0	87	9	586	1,15	0,00	0,36	0,35	1,33	0,24817	0,89634
	hPIV-2/4	0	16	9	657	0,21	0,00	2,07	2,10	0,22	0,63972	0,89634
	HAdV	1	88	8	585	1,17	0,85	0,83	0,83	0,03	0,86201	0,89634
	HBoV	0	43	9	630	0,57	0,00	0,77	0,76	0,61	0,43339	0,89634
	HCoV	0	54	9	619	0,71	0,00	0,60	0,60	0,78	0,37585	0,89634
	HMPV	0	55	9	618	0,73	0,00	0,59	0,59	0,80	0,37108	0,89634
	RSV	1	102	8	571	1,36	0,74	0,70	0,70	0,11	0,73638	0,89634
	HRV	8	269	1	404	3,66	2,19	11,70	12,01	8,81	0,00299	0,06842
Certain lower respiratory tract diseases (CA20-CA2Z)	EV	1	19	6	656	0,21	4,87	5,52	5,75	3,20	0,07352	0,71296
	IV-A/B	0	34	7	641	0,35	0,00	1,24	1,24	0,37	0,54241	0,89634
	hPIV-1/3	1	86	6	589	0,89	1,12	1,14	1,14	0,01	0,90298	0,91153
	hPIV-2/4	0	16	7	659	0,16	0,00	2,62	2,66	0,17	0,68019	0,89634
	HAdV	3	86	4	589	0,91	3,28	5,00	5,14	5,54	0,01861	0,23819
	HBoV	0	43	7	632	0,44	0,00	0,97	0,97	0,48	0,49027	0,89634
	HCoV	1	53	6	622	0,55	1,80	1,94	1,96	0,39	0,53055	0,89634
	HMPV	0	55	7	620	0,56	0,00	0,75	0,75	0,62	0,43090	0,89634

Disease categories	Viral group	a	b	c	d	Expected count	Lift	Risk ratio (RR)	Odds ratio (OR)	χ²	p-value	q-value (Benjamini-Hochberg)
	RSV	0	103	7	572	1,06	0,00	0,37	0,37	1,26	0,26200	0,89634
	HRV	1	276	6	399	2,84	0,35	0,24	0,24	2,03	0,15393	0,89634
Viral infections characterized by skin or mucous membrane lesions (1F00-1F0Z)	EV	0	20	5	657	0,15	0,00	2,87	2,92	0,15	0,69647	0,89634
	IV-A/B	0	34	5	643	0,25	0,00	1,69	1,70	0,26	0,60719	0,89634
	hPIV-1/3	0	87	5	590	0,64	0,00	0,62	0,61	0,74	0,39079	0,89634
	hPIV-2/4	0	16	5	661	0,12	0,00	3,57	3,64	0,12	0,72794	0,89634
	HAdV	0	89	5	588	0,65	0,00	0,60	0,60	0,76	0,38459	0,89634
	HBoV	0	43	5	634	0,32	0,00	1,32	1,33	0,34	0,56044	0,89634
	HCoV	0	54	5	623	0,40	0,00	1,04	1,04	0,43	0,51047	0,89634
	HMPV	0	55	5	622	0,40	0,00	1,02	1,02	0,44	0,50624	0,89634
	RSV	1	102	4	575	0,76	1,32	1,41	1,41	0,09	0,75888	0,89634
	HRV	4	273	1	404	2,03	1,97	5,85	5,92	3,24	0,07189	0,71296
Certain staphylococcal or streptococcal diseases (1B40-1B5Z)	EV	0	20	4	658	0,12	0,00	3,51	3,57	0,12	0,72735	0,89634
	IV-A/B	0	34	4	644	0,20	0,00	2,06	2,08	0,21	0,64590	0,89634
	hPIV-1/3	0	87	4	591	0,51	0,00	0,75	0,75	0,59	0,44307	0,89634
	hPIV-2/4	0	16	4	662	0,09	0,00	4,36	4,46	0,10	0,75587	0,89634
	HAdV	0	89	4	589	0,52	0,00	0,73	0,73	0,60	0,43710	0,89634
	HBoV	1	42	3	636	0,25	3,97	4,95	5,05	2,38	0,12286	0,89634
	HCoV	1	53	3	625	0,32	3,16	3,88	3,93	1,61	0,20445	0,89634
	HMPV	1	54	3	624	0,32	3,10	3,80	3,85	1,56	0,21218	0,89634
	RSV	0	103	4	575	0,60	0,00	0,62	0,62	0,72	0,39754	0,89634
	HRV	1	276	3	402	1,62	0,62	0,49	0,49	0,41	0,52360	0,89634
Primary immunodeficiencies (4A00-4A0Z)	EV	0	20	3	659	0,09	0,00	4,51	4,60	0,09	0,76287	0,89634
	IV-A/B	0	34	3	645	0,15	0,00	2,65	2,67	0,16	0,69091	0,89634
	hPIV-1/3	2	85	1	594	0,38	5,23	13,68	13,98	7,87	0,00503	0,09467
	hPIV-2/4	1	15	2	664	0,07	14,21	20,81	22,13	12,63	0,00038	0,02025
	HAdV	0	89	3	590	0,39	0,00	0,94	0,94	0,45	0,50127	0,89634
	HBoV	2	41	1	638	0,19	10,57	29,72	31,12	18,58	0,00002	0,00173
	HCoV	1	53	2	626	0,24	4,21	5,81	5,91	2,67	0,10228	0,83923
	HMPV	0	55	3	624	0,24	0,00	1,60	1,61	0,26	0,60717	0,89634
	RSV	1	102	2	577	0,45	2,21	2,81	2,83	0,78	0,37681	0,89634
	HRV	0	277	3	402	1,22	0,00	0,21	0,21	2,06	0,15112	0,89634

Table S3. Statistical parameters of the local association analysis between nosological forms and viral groups based on 2×2 contingency tables.

Nosological form	Viral group	a	b	c	d	Expected count	Lift	Risk ratio (RR)	Odds ratio (OR)	χ²	p-value	q-value (Benjamini-Hochberg)
Acute upper respiratory infections of multiple or unspecified sites (CA07)	IV-A/B	27	203	7	406	12,16	2,220	6,926	7,714	29,75	4,89E-08	4,89E-06
	HAdV	31	199	52	361	29,69	1,044	1,070	1,081	0,103	0,74767	0,90081
	HBoV	10	220	34	379	15,74	0,635	0,528	0,507	3,497	0,06148	0,32356
	RSV	20	210	79	334	35,41	0,565	0,455	0,403	12,34	0,00044	0,00885
	HMPV	18	212	34	379	18,60	0,968	0,951	0,946	0,033	0,85624	0,92945

Nosological form	Viral group	a	b	c	d	Expected count	Lift	Risk ratio (RR)	Odds ratio (OR)	χ^2	p-value	q-value (Benjamini-Hochberg)
	hPIV-2/4	7	223	9	404	5,72	1,223	1,397	1,409	0,455	0,50006	0,83441
	hPIV-1/3	32	198	50	363	29,33	1,091	1,149	1,173	0,433	0,51037	0,83441
	HRV	94	136	165	248	92,64	1,015	1,023	1,039	0,052	0,82004	0,92945
	EV	7	223	12	401	6,80	1,030	1,047	1,049	0,010	0,92115	0,94973
	HCoV	15	215	34	379	17,53	0,856	0,792	0,778	0,614	0,43324	0,83441
Lower respiratory tract diseases (bronchitis/ bronchiolitis) (CA42, CA43)	IV-A/B	1	131	33	478	6,98	0,143	0,117	0,111	6,806	0,00908	0,06488
	HAdV	13	119	70	441	17,04	0,763	0,719	0,688	1,383	0,23955	0,64744
	HBoV	8	124	36	475	9,03	0,886	0,860	0,851	0,159	0,68965	0,88345
	RSV	29	103	70	441	20,32	1,427	1,604	1,774	5,509	0,01892	0,12611
	HMPV	13	119	39	472	10,67	1,218	1,290	1,322	0,693	0,40505	0,83441
	hPIV-2/4	3	129	13	498	3,28	0,913	0,893	0,891	0,032	0,85842	0,92945
	hPIV-1/3	14	118	68	443	16,83	0,832	0,797	0,773	0,688	0,40687	0,83441
	HRV	56	76	203	308	53,17	1,053	1,068	1,118	0,317	0,57312	0,83441
	EV	3	129	16	495	3,90	0,769	0,726	0,719	0,270	0,60364	0,84246
	HCoV	12	120	37	474	10,06	1,193	1,256	1,281	0,510	0,47509	0,83441
Pneumonia (CA40)	IV-A/B	1	131	33	478	6,98	0,143	0,117	0,111	6,806	0,00908	0,06488
	HAdV	8	124	75	436	17,04	0,470	0,413	0,375	6,928	0,00849	0,06488
	HBoV	16	116	28	483	9,03	1,771	2,212	2,379	7,259	0,00705	0,06412
	RSV	35	97	64	447	20,32	1,722	2,117	2,520	15,76	0,00007	0,00179
	HMPV	14	118	38	473	10,67	1,311	1,426	1,477	1,418	0,23375	0,64744
	hPIV-2/4	1	131	15	496	3,28	0,304	0,258	0,252	2,051	0,15215	0,53077
	hPIV-1/3	17	115	65	446	16,83	1,010	1,012	1,014	0,002	0,96115	0,97086
	HRV	57	75	202	309	53,17	1,072	1,092	1,163	0,581	0,44574	0,83441
	EV	1	131	18	493	3,90	0,256	0,215	0,209	2,797	0,09446	0,42937
	HCoV	4	128	45	466	10,06	0,398	0,344	0,324	4,971	0,02577	0,15159
Acute laryngitis or tracheitis (CA23)	IV-A/B	1	31	33	578	1,69	0,591	0,579	0,565	0,315	0,57492	0,83441
	HAdV	3	29	80	531	4,13	0,726	0,716	0,687	0,374	0,54085	0,83441
	HBoV	3	29	41	570	2,19	1,370	1,397	1,438	0,339	0,56058	0,83441
	RSV	3	29	96	515	4,93	0,609	0,597	0,555	0,937	0,33295	0,79273
	HMPV	1	31	51	560	2,59	0,386	0,374	0,354	1,116	0,29088	0,72721
	hPIV-2/4	2	30	14	597	0,80	2,512	2,728	2,843	1,964	0,16110	0,53700
	hPIV-1/3	7	25	75	536	4,08	1,715	1,782	2,001	2,519	0,11251	0,45724
	HRV	12	20	247	364	12,89	0,931	0,928	0,884	0,108	0,74222	0,90081
	EV	1	31	18	593	0,95	1,058	1,061	1,063	0,003	0,95352	0,97086
	HCoV	3	29	46	565	2,44	1,230	1,245	1,271	0,147	0,70118	0,88345
Fever of other or unknown origin (MG26)	IV-A/B	0	28	34	581	1,48	0,000	0,000	0,000	1,634	0,20110	0,59146
	HAdV	4	24	79	536	3,61	1,107	1,112	1,131	0,049	0,82409	0,92945
	HBoV	1	27	43	572	1,92	0,522	0,511	0,493	0,492	0,48326	0,83441
	RSV	4	24	95	520	4,31	0,928	0,925	0,912	0,028	0,86774	0,92945
	HMPV	2	26	50	565	2,26	0,883	0,879	0,869	0,035	0,85136	0,92945
	hPIV-2/4	1	27	15	600	0,70	1,435	1,464	1,481	0,142	0,70676	0,88345
	hPIV-1/3	3	25	79	536	3,57	0,840	0,834	0,814	0,109	0,74091	0,90081
	HRV	7	21	252	363	11,28	0,621	0,610	0,480	2,841	0,09187	0,42937
	EV	1	27	18	597	0,83	1,209	1,220	1,228	0,039	0,84384	0,92945

Nosological form	Viral group	a	b	c	d	Expected count	Lift	Risk ratio (RR)	Odds ratio (OR)	χ^2	p-value	q-value (Benjamini-Hochberg)
	HCoV	5	23	44	571	2,13	2,343	2,496	2,821	4,358	0,03684	0,20469
Acute nasopharyngitis (CA00)	IV-A/B	2	24	32	585	1,37	1,455	1,483	1,523	0,313	0,57595	0,83441
	HAdV	9	17	74	543	3,36	2,682	2,886	3,885	11,35	0,00075	0,01074
	HBoV	1	25	43	574	1,78	0,562	0,552	0,534	0,382	0,53668	0,83441
	RSV	3	23	96	521	4,00	0,749	0,742	0,708	0,310	0,57791	0,83441
	HMPV	1	25	51	566	2,10	0,476	0,465	0,444	0,656	0,41811	0,83441
	hPIV-2/4	1	25	15	602	0,65	1,546	1,582	1,605	0,206	0,65002	0,87840
	hPIV-1/3	4	22	78	539	3,32	1,206	1,217	1,256	0,169	0,68128	0,88345
	HRV	5	21	254	363	10,47	0,477	0,467	0,340	4,991	0,02548	0,15159
	EV	1	25	18	599	0,77	1,302	1,318	1,331	0,075	0,78411	0,92249
	HCoV	2	24	47	570	1,98	1,009	1,010	1,011	0,000	0,98876	0,98876
Acute tonsillitis (CA03)	IV-A/B	0	23	34	586	1,22	0,000	0,000	0,000	1,332	0,24850	0,65395
	HAdV	8	15	75	545	2,97	2,695	2,875	3,876	10,15	0,00144	0,01801
	HBoV	0	23	44	576	1,57	0,000	0,000	0,000	1,752	0,18561	0,56244
	RSV	1	22	98	522	3,54	0,282	0,275	0,242	2,235	0,13488	0,51877
	HMPV	0	23	52	568	1,86	0,000	0,000	0,000	2,099	0,14742	0,53077
	hPIV-2/4	0	23	16	604	0,57	0,000	0,000	0,000	0,609	0,43528	0,83441
	hPIV-1/3	2	21	80	540	2,93	0,682	0,674	0,643	0,353	0,55249	0,83441
	HRV	8	15	251	369	9,26	0,864	0,859	0,784	0,300	0,58409	0,83441
	EV	4	19	15	605	0,68	5,886	7,188	8,491	17,33	0,00003	0,00130
	HCoV	2	21	47	573	1,75	1,141	1,147	1,161	0,039	0,84312	0,92945
Otitis media (AB00)	IV-A/B	1	11	33	598	0,63	1,576	1,593	1,647	0,226	0,63414	0,86869
	HAdV	5	7	78	553	1,55	3,228	3,371	5,064	8,996	0,00271	0,03006
	HBoV	2	10	42	589	0,82	2,436	2,504	2,805	1,851	0,17364	0,56013
	RSV	1	11	98	533	1,85	0,541	0,537	0,494	0,468	0,49375	0,83441
	HMPV	0	12	52	579	0,97	0,000	0,000	0,000	1,076	0,29961	0,73076
	hPIV-2/4	0	12	16	615	0,30	0,000	0,000	0,000	0,312	0,57643	0,83441
	hPIV-1/3	0	12	82	549	1,53	0,000	0,000	0,000	1,787	0,18125	0,56244
	HRV	5	7	254	377	4,83	1,034	1,035	1,060	0,010	0,92124	0,94973
	EV	1	11	18	613	0,35	2,820	2,921	3,096	1,234	0,26672	0,68389
	HCoV	2	10	47	584	0,91	2,187	2,238	2,485	1,421	0,23317	0,64744
Primary immunodeficiencies (4A00-4A0Z)	IV-A/B	0	3	34	606	0,16	0,000	0,000	0,000	0,168	0,68165	0,88345
	HAdV	0	3	83	557	0,39	0,000	0,000	0,000	0,447	0,50389	0,83441
	HBoV	2	1	42	598	0,21	9,742	10,15	28,47	16,92	0,00004	0,00130
	RSV	1	2	98	542	0,46	2,165	2,177	2,765	0,744	0,38824	0,83441
	HMPV	0	3	52	588	0,24	0,000	0,000	0,000	0,265	0,60657	0,84246
	hPIV-2/4	1	2	15	625	0,07	13,39	14,22	20,83	11,81	0,00059	0,00978
	hPIV-1/3	2	1	80	560	0,38	5,228	5,333	14,0	7,874	0,00501	0,05015
	HRV	0	3	259	381	1,21	0,000	0,000	0,000	2,033	0,15392	0,53077
	EV	0	3	19	621	0,09	0,000	0,000	0,000	0,092	0,76193	0,90706
	HCoV	1	2	48	592	0,23	4,374	4,444	6,167	2,831	0,09248	0,42937

Table S4. ICD-11 Diagnoses Included in the Disease Categories Used for Association Analysis

Disease category	Diagnoses included	ICD-11 codes
Upper respiratory tract diseases	Acute nasopharyngitis; Acute pharyngitis, unspecified; Acute tonsillitis, unspecified; Acute laryngitis; Acute tracheitis; Acute laryngotracheitis; Acute upper respiratory infections of multiple and unspecified sites; Chronic rhinitis, nasopharyngitis or pharyngitis; Chronic rhinitis	CA00; CA02.Z; CA03.Z; CA05.0; CA05.1; CA05.2; CA07; CA09; CA09.0
Pulmonary infections	Pneumonia due to *Mycoplasma pneumoniae*; Pneumonia due to *Streptococcus pneumoniae*; Pneumonia due to other specified bacteria; Pneumonia due to respiratory syncytial virus; Pneumonia due to parainfluenza virus; Pneumonia due to other specified virus; Viral pneumonia, unspecified; Pneumonia, organism unspecified; Acute bronchiolitis due to respiratory syncytial virus; Other specified acute bronchiolitis; Acute bronchiolitis, unspecified; Acute bronchitis due to rhinovirus; Acute bronchitis due to respiratory syncytial virus; Other specified acute bronchitis; Acute bronchitis, unspecified	CA40.04; CA40.07; CA40.0Y; CA40.11; CA40.12; CA40.1Y; CA40.1Z; CA40.Z; CA41.0; CA41.Y; CA41.Z; CA42.1; CA42.2; CA42.Y; CA42.Z
Symptoms, signs or clinical findings of the respiratory system	Cough	MD12
Other bacterial diseases	Whooping cough, unspecified; Meningococcaemia; Other specified meningococcal disease; Bacterial infection of unspecified site	1C12.Z; 1C1C.2; 1C1C.Y; 1C41
Diseases of middle ear or mastoid	Nonsuppurative otitis media, unspecified; Acute suppurative otitis media	AA8Z; AA90
General symptoms, signs or clinical findings	Feeling ill; Fever of other or unknown origin	MG25; MG26
Respiratory disorders specific to the perinatal or neonatal period	Congenital pneumonia; Bronchopulmonary dysplasia originating in the perinatal period; Other specified respiratory disorders specific to the perinatal or neonatal period	KB24; KB29.0; KB2Y
Certain lower respiratory tract diseases	Acute pharyngitis, unspecified; Chronic obstructive pulmonary disease, unspecified; Cystic fibrosis	CA02.Z; CA22.Z; CA25
Viral infections characterised by skin or mucous membrane lesions	Varicella encephalitis; Varicella with other specified complication; Varicella, unspecified; Measles with other complications; Viral infections characterised by skin or mucous membrane lesions, unspecified	1E90.2; 1E90.Y; 1E90.Z; 1F03.Y; 1F0Z
Certain staphylococcal or streptococcal diseases	Scarlet fever; Meningitis due to *Streptococcus*; Staphylococcal or streptococcal diseases, unspecified	1B50; 1B53; 1B5Z
Primary immunodeficiencies	Combined immunodeficiencies, unspecified; Other specified primary immunodeficiencies; Primary immunodeficiencies, unspecified	4A01.1Z; 4A0Y; 4A0Z
