# Supplementary material for: Etiology and Clinical Manifestations of Acute Respiratory Infections in Pediatric Patients: A Retrospective Study in Moscow, Russia
Source: Diseases. 2026 Sep 6;14(9):326. doi: 10.3390/diseases14090326 (PMC13605983; doi:10.3390/diseases14090326)
Supplement: Supplementary file 1 [file diseases-14-00326-s001.zip › Table S5 STROBE-checklist_ARI-MDPI-23.08.2026.pdf]

Table S5. STROBE Statement Checklist

Etiology and Clinical Manifestations of Acute Respiratory Infections in Pediatric Patients: A Retrospective Study in Moscow, Russia

Section	Item No.	STROBE recommendation	Page/line and relevant manuscript information
**Title and abstract**	**1a**	Indicate the study design with a commonly used term in the title or abstract.	The manuscript is identified as a retrospective, single-center observational study.
	**1b**	Provide an informative and balanced summary of what was done and what was found.	The abstract reports the study setting, sample size, molecular testing approach, principal etiological associations, severity findings, and conclusions.
**Introduction**	**2**	Explain the scientific background and rationale for the investigation.	Acute respiratory infections (ARIs) are a leading cause of pediatric morbidity worldwide, with clinical presentations ranging from mild upper respiratory illness to severe lower respiratory tract disease requiring hospitalization. The clinical presentation of ARIs is notably heterogeneous, ranging from mild, self-limiting upper respiratory tract involvement to severe lower respiratory tract disease requiring hospitalization and intensive care. Effective epidemiological surveillance of respiratory viruses is essential for monitoring disease trends, detecting emerging pathogens, and informing public health interventions. Real-time PCR has enhanced pathogen detection, yet challenges persist in distinguishing active infection from incidental viral shedding and interpreting the clinical relevance of co-infections. Broad-spectrum screening panels now enable simultaneous detection of multiple respiratory pathogens, providing valuable insights into the viral etiology of ARIs. However, the prognostic value of specific viral pathogens in predicting disease severity is not fully established, and the clinical significance of detecting multiple viruses in a single patient remains challenging to interpret.
	**3**	State the specific objectives, including any prespecified hypotheses.	The primary objective of this study was to evaluate the clinical and etiological associations of acute respiratory infections (ARIs) in a pediatric cohort, with a particular focus on identifying factors related to nosological form and disease severity.
**Methods**	**4**	Present key elements of the study design early in the paper.	Section 2.1. Study design: retrospective, single-center observational study.
	**5**	Describe the setting, locations, and relevant dates, including periods of data collection.	Section 2.1. Samples were collected at G. N. Speransky Children's City Clinical Hospital No. 9 from 20 September 2021 to 31 July 2024.
	**6a**	Give eligibility criteria and the sources and methods of selection of participants.	Section 2.1. PCR testing was performed in pediatric patients with ARI manifestations or respiratory symptoms identified during clinical evaluation. The analyzed cohort was restricted to non-COVID-19 ARI cases.
	**6b**	For matched studies, give matching criteria and numbers in each group.	Not applicable. The study did not use a matched design.
	**7**	Clearly define outcomes, exposures, predictors, potential confounders, and diagnostic criteria where applicable.	Sections 2.1-2.4. The manuscript defines clinical metadata, viral groups, ICD-11 disease categories and nosological forms, severe disease criteria, age groups, and co-infection variables.
	**8**	For each variable of interest, give sources of data and details of measurement or assessment methods.	Sections 2.1-2.2. Clinical metadata were obtained from medical records; respiratory viruses were identified by real-time PCR using specified commercial assays and manufacturer-defined Ct criteria; disease severity criteria are explicitly described.
	**9**	Describe any efforts to address potential sources of bias.	Section 2.1; Discussion. All eligible observations from the predefined period were included, PCR testing used assay controls and predefined positivity criteria, and selection, confounding, specimen-related, and diagnostic limitations are discussed.
	**10**	Explain how the study size was arrived at.	Section 2.1. The study size was determined by all observations meeting eligibility criteria during the predefined period; no a priori sample-size calculation was performed.
	**11**	Explain how quantitative variables were handled. If variables were grouped, describe the group boundaries and why they were chosen.	Section 2.4. Age was categorized using predefined non-overlapping intervals. Finer categories were retained during the first year of life to preserve age-related resolution in infancy.
	**12a**	Describe all statistical methods, including those used to control for confounding.	Sections 2.3-2.4. The manuscript describes contingency-table analyses, chi-square testing, standardized residuals, Lift, ORs with 95% CIs, FDR correction, Wilson intervals, and logistic regression. ORs are identified as unadjusted; multivariable adjustment was not performed because detailed clinical covariates were not consistently available.
	**12b**	Describe methods used to examine subgroups and interactions.	Section 2.4. Age-stratified severity analyses and analyses of viral co-infections are described.

Section	Item No.	STROBE recommendation	Page/line and relevant manuscript information
	**12c**	Explain how missing data were addressed.	Section 2.4. Age was unavailable for two observations. These cases were retained for all analyses not involving age and excluded from age-stratified analyses. No imputation was performed.
	**12d**	If applicable, explain how loss to follow-up, matching, or a complex sampling strategy was addressed.	Not applicable. The study was retrospective and did not involve prospective follow-up, matching, or a complex sampling design requiring weighting.
	**12e**	Describe any sensitivity analyses.	Not applicable. No sensitivity analyses were performed.
**Results**	**13a**	Report numbers of individuals or observations at each relevant stage of the study.	Section 3.1. The final analysis included 1,362 pediatric ARI cases.
	**13b**	Give reasons for non-participation or exclusion at each stage.	Section 2.1. The analyzed dataset was restricted to non-COVID-19 ARI cases.
	**13c**	Consider use of a flow diagram.	Not used. Participant selection was straightforward and is described in the text; all eligible observations from the predefined period were included.
	**14a**	Give characteristics of study participants and information on relevant exposures and potential confounders.	Section 3.1, The demographic and clinical characteristics of the study cohort are summarized in Figure 1.
	**14b**	Indicate the number of participants with missing data for each variable of interest.	Section 2.4. Among all variables of interest, missing data were only identified for age (n = 2). No other missing values were present for sex, clinical department, hospitalization status, or specimen type. The two cases with incomplete age records were retained in analyses not involving age.
	**14c**	For cohort studies, summarize follow-up time.	Not applicable. No longitudinal follow-up period was analyzed.
	**15**	Report numbers of outcome events or summary measures over time.	Sections 3.2 and 3.5-3.7, Figures 2 and 5. Viral detection frequencies, severe disease proportions, age-related severity, and co-infection outcomes are reported.
	**16a**	Give unadjusted estimates and, if applicable, confounder-adjusted estimates with measures of precision.	Sections 3.3-3.7; Figures 3-5. ORs are presented with 95% confidence intervals and relevant p or q values. Section 2.3 states that reported ORs are unadjusted.
	**16b**	When continuous variables are categorized, report category boundaries.	Section 2.4. The boundaries of all age categories are explicitly stated.
	**16c**	If relevant, consider translating estimates of relative risk into absolute risk for a meaningful period.	Sections 3.5-3.7; Figure 5. Absolute proportions of severe cases with 95% confidence intervals are reported together with OR estimates.
	**17**	Report other analyses performed, for example subgroup analyses and sensitivity analyses.	Sections 3.6-3.7; Figure 5B-E. Age-stratified analyses, virus-by-age severity estimates, and co-infection analyses are reported.
**Discussion**	**18**	Summarize key results with reference to the study objectives.	Discussion, pp. 13-16. The Discussion summarizes the principal etiological associations, severity findings, age effects, and co-infection results in relation to the study objective.
	**19**	Discuss limitations, taking into account sources of potential bias or imprecision, including their direction and magnitude.	Discussion, p.16. Key limitations include the retrospective single-center design, unavailability of clinical confounders, ICD-11-based diagnosis categorization, hospital-based patient selection, predominant use of oropharyngeal swabs, incomplete etiological verification, and lack of bacteriological or serological data. The potential impact of these biases could not be assessed quantitatively.
	**20**	Give a cautious overall interpretation considering objectives, limitations, multiplicity of analyses, similar studies, and other relevant evidence.	Discussion, pp. 13-16. Interpretation of the findings is contextualized by published pediatric respiratory-virus data, with due caution applied to small subgroup sizes, co-infection dynamics, selection bias, and the inherent limitations of etiological verification.
	**21**	Discuss the generalisability of the study results.	Discussion, p. 16. The findings of this study should be interpreted in light of the specific clinical and methodological context in which they were obtained. Given the retrospective, single-center design and the hospital-based nature of the cohort, the observed etiological distribution and severity profile may not be directly generalizable to community-based pediatric populations or to settings with different hospitalization practices, patient referral patterns, or molecular diagnostic protocols. In particular, the study population likely includes a higher proportion of moderate-to-severe cases compared with outpatient or community cohorts, which may overestimate the prevalence of certain viral pathogens and severity associations.
**Other information**	**22**	Give the source of funding and describe the role of the funders for the present study.	Author Contributions and Funding. FMBA is identified as the funding source. The Author Contributions statement specifies the scientific roles of the investigators, including FMBA-affiliated authors, in conceptualization, methodology, analysis, writing, supervision, project administration, and funding acquisition.
